# Atypical Teratoid Rhabdoid Tumours Are Susceptible to Panobinostat-Mediated Differentiation Therapy

**DOI:** 10.3390/cancers13205145

**Published:** 2021-10-14

**Authors:** Wai C. Chong, W. Samantha N. Jayasekara, Vijesh G. Vaghjiani, Sarah Parackal, Claire Sun, Dean Popovski, Elizabeth M. Algar, Ron Firestein, Paul J. Wood, Sara Khan, Annie Huang, David M. Ashley, Peter Downie, Jason E. Cain

**Affiliations:** 1 Centre for Cancer Research, Hudson Institute of Medical Research, Clayton, VIC 3168, Australia; waichin.chong@hudson.org.au (W.C.C.); samantha.jayasekara@monash.edu (W.S.N.J.); vijesh.vaghjiani@hudson.org.au (V.G.V.); sarah.parackal@monash.edu (S.P.); claire.sun@hudson.org.au (C.S.); dean.popovski@anu.edu.au (D.P.); elizabeth.algar@monash.edu (E.M.A.); ron.firestein@hudson.org.au (R.F.); sara.khan@sickkids.ca (S.K.); 2 Department of Molecular and Translational Sciences, Monash University, Clayton, VIC 3168, Australia; 3 Children’s Cancer Centre, Monash Children’s Hospital, Monash Health, Clayton, VIC 3168, Australia; paul.wood@monashhealth.org (P.J.W.); Peter.Downie@monashhealth.org (P.D.); 4 Department of Paediatrics, Monash University, Clayton, VIC 3168, Australia; 5 Arthur and Sonia Labatt Brain Tumor Research Centre, Division of Haematology/Oncology, Hospital for Sick Children, Toronto, ON M5G 1X8, Canada; annie.huang@sickkids.ca; 6 Laboratory of Medicine and Pathobiology, Department of Pediatrics, Medical Biophysics, Faculty of Medicine, University of Toronto, Toronto, ON M5S 1A8, Canada; 7 Division of Hematology-Oncology, Department of Pediatrics, The Hospital for Sick Children, University of Toronto, Toronto, ON M5G 1X8, Canada; 8 The Preston Robert Tisch Brain Tumor Centre at Duke, Duke University Medical Centre, Durham, NC 27710, USA; david.ashley@duke.edu

**Keywords:** atypical teratoid rhabdoid tumour, panobinostat, HDACi, differentiation

## Abstract

**Simple Summary:**

Atypical teratoid rhabdoid tumour (ATRT) is an aggressive undifferentiated malignancy of the central nervous system in children. A defining feature of ATRT is the loss of the *SMARCB1* gene that is essential for regulating gene expression required for normal developmental processes. We show that treatment of human ATRT cell models with the histone deacetylate inhibitor, panobinostat, inhibits tumour growth, reactivates the expression of developmental genes, and drives neuronal differentiation. These results demonstrate the therapeutic potential of panobinostat for the treatment of ATRT.

**Abstract:**

Atypical teratoid rhabdoid tumour (ATRT) is a rare but highly aggressive undifferentiated solid tumour arising in the central nervous system and predominantly affecting infants and young children. ATRT is exclusively characterized by the inactivation of *SMARCB1*, a member of the SWI/SNF chromatin remodelling complex that is essential for the regulation of large sets of genes required for normal development and differentiation. Histone deacetylase inhibitors (HDACi) are a promising anticancer therapy and are able to mimic the normal acetylation functions of SMARCB1 in *SMARCB1*-deficient cells and drive multilineage differentiation in extracranial rhabdoid tumours. However, the potential efficacy of HDACi in ATRT is unknown. Here, we show that human ATRT cells are highly responsive to the HDACi panobinostat and that sustained treatment leads to growth arrest, increased cell senescence, decreased clonogenicity and induction of a neurogenesis gene-expression profile. Furthermore, in an orthotopic ATRT xenograft model, continuous panobinostat treatment inhibits tumour growth, increases survival and drives neuronal differentiation as shown by the expression of the neuronal marker, TUJ1. Collectively, this preclinical study supports the therapeutic potential of panobinostat-mediated differentiation therapy for ATRT.

## 1. Introduction

Intracranial malignant rhabdoid tumours, termed atypical teratoid rhabdoid tumours (ATRT), are aggressive, undifferentiated malignancies with poor prognosis [1,2]. ATRT accounts for 1–2% of CNS tumours in children, with the majority occurring in children under three years [2]. Like extracranial malignant rhabdoid tumours, ATRT is genetically characterized by homozygous inactivating mutations in the *SWItch/Sucrose Non-Fermentable (SWI/SNF)-related*, *Matrix-associated*, *actin-dependent Regulator of Chromatin*, *subfamily B*, *member 1* (*SMARCB1)* gene, or less frequently *SMARCA4*, which encode core subunits of the SWI/SNF chromatin remodelling complex [3]. Importantly, it is also characterized by a remarkably stable genome, having the lowest base rate change among all sequenced malignancies, suggesting that *SMARCB1* inactivation alone is sufficient to initiate ATRT tumourigenesis and that epigenetic dysregulation plays a significant role in the pathogenesis of the disease [4,5,6].

The current standard of care for ATRT includes surgery, radiotherapy and chemotherapy. Recent investigations have identified age less than one year and DNA methylation as independent risk factors, with the idea of more targeted treatment stratification [7]. Complete tumour resection provides the best chance of cure but is often impractical due to the location of the tumour. Indeed, the Canadian Brain Tumour Consortium showed gross total resection (GTR) to be achievable in around 30% of patients, with a median survival of the whole cohort of 13.5 months. Those patients who achieved GTR had superior outcomes [8]. Irradiation is not recommended in patients less than three years of age, the most predominant age group for ATRT, due to severe long-term neurocognitive and neuroendocrine consequences [9]. The use of high-dose chemotherapy is associated with better responses but contributes to significant systemic toxicity [2,10]. Because of these factors, current therapy is largely ineffective, with an estimated two- and five-year overall survival of 42% and 28%, respectively [11], highlighting the urgent need for improved therapeutic options for ATRT. Rhabdoid tumours exhibit hypo-acetylated chromatin, suggesting that SMARCB1-mediated SWI/SNF functions are essential for histone acetylation, thereby implicating insufficient histone acetylation as an underlying pathogenic mechanism in SMARCB1-deficient ATRT. Indeed, pan-histone deacetylase inhibitors (HDACi), such as romidepsin, cyclic hydroxamic-acid-containing peptide 31 and panobinostat, are able to mimic the histone acetylation function of SWI/SNF complexes in SMARCB1-deficient cells [12,13,14], implicating HDACi as compounds of interest warranting further investigation in ATRT.

ATRT is characterized by a primitive undifferentiated pathology and a stem cell-like gene expression profile [15,16]. Since SMARCB1 and the SWI/SNF complex are required for lineage commitment and differentiation during normal development, loss of SMARCB1 in ATRT is proposed to contribute to the maintenance of the undifferentiated phenotype [17,18,19] and suggests that ATRT may be amendable to differentiation therapy. The differentiation potential of HDACi, such as panobinostat, vorinostat or trichostatin A has been described in normal adipocyte development, mesenchymal stem cells, and undifferentiated myeloid malignancies [20,21,22]. Moreover, our previous work with panobinostat in osteosarcoma and extracranial rhabdoid tumour demonstrate the ability of sustained low-dose HDACi to inhibit tumour growth and drive multi-lineage cellular differentiation in solid malignancies [23,24].

Here, for the first time, we explored the therapeutic potential of epigenetic differentiation therapy in ATRT. Human ATRT cell lines are highly responsive to the HDACi panobinostat, and sustained treatment with a low, non-cytotoxic dose, leads to growth arrest and gene expression changes consistent with neuronal differentiation. Furthermore, continuous panobinostat treatment of an orthotopic ATRT xenograft model inhibits tumour growth, increases survival and drives expression of the neuronal-specific Class III Beta Tubulin (TUJ1). Since current treatment approaches for ATRT are limited, low-dose panobinostat may have clinical potential as a non-cytotoxic epigenetic differentiation therapy.

## 2. Materials and Methods

### 2.1. Cell Lines and Culture Conditions

**Figure 1:**
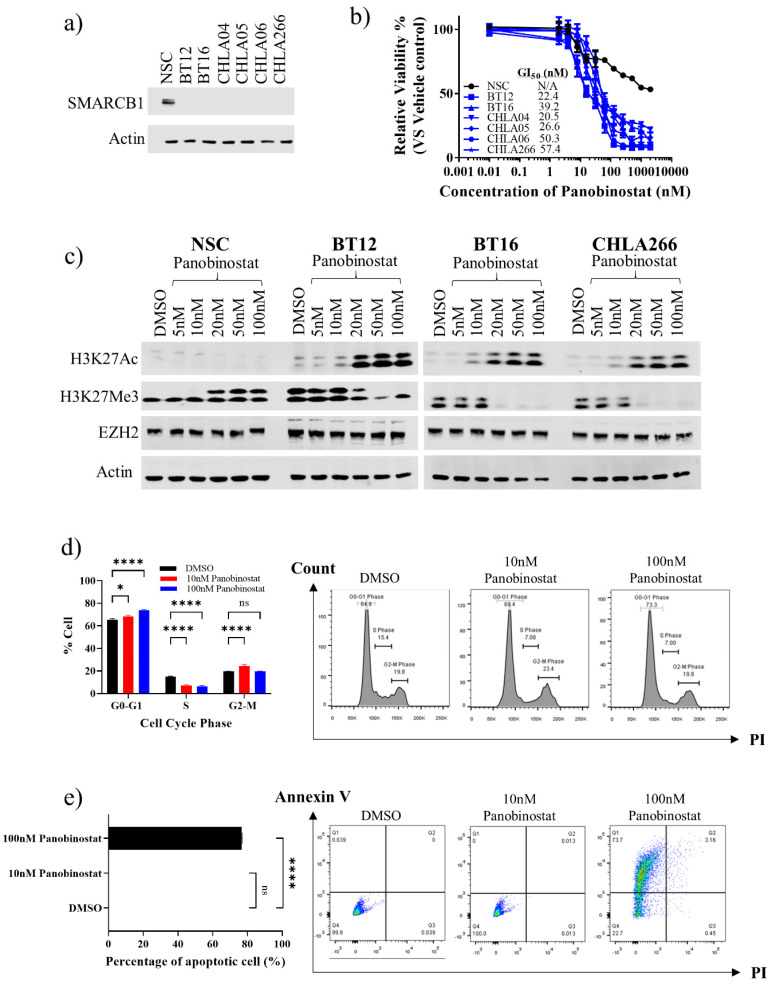
Response of human ATRT cell lines to the HDACi, panobinostat, following 72 h of treatment. (**a**) Analysis of SMARCB1 protein expression in human ATRT cell lines (BT12, BT16, CHLA04, CHLA05, CHLA06 and CHLA266) and human NSC. (**b**) Cell viability of ATRT cell lines and NSC treated with increasing concentrations of panobinostat (0.01–2000 nM). (*n* = 4; mean ± SEM). (**c**) Western blot of histone H3 acetylation (H3K27Ac), methylation (H3K27Me3), EZH2 and Actin in SMARCB1-null ATRT cells (BT12, BT16, and CHLA266) and NSC cells after treatment with increasing doses of panobinostat (5–100 nM) for 72 h. (**d**) Cell cycle analysis of BT12 treated with increasing doses of panobinostat (10–100 nM) for 72 h (*n* = 3; mean ± SEM; ns, not significant; *, *p* < 0.05; ****, *p* < 0.0001) (**e**) Annexin V assay of BT12 treated with increasing doses of panobinostat (10–100 nM) for 72 h (*n* = 3; mean ± SEM; ns, not significant; ****, *p* < 0.0001). Apoptotic cells are defined as Annexin V +ve, PI −ve and Annexin V +ve, PI −ve.

Authenticated ATRT cell lines BT-12 and BT-16 cell lines were provided by Dr Peter Houghton (St. Jude Children’s Research Hospital), CHLA266 was obtained from the Childhood Cancer Repository and CHLA04, CHLA05 and CHLA06 were obtained from the American Type Culture Collection (ATCC). CHLA04 and CHLA05 belong to the ATRT-SHH subgroup while BT12, BT16, CHLA06 and CHLA266 belong to the ATRT-MYC subgroup [25]. All cell lines were re-authenticated in our laboratory by short tandem repeat analysis (STR) immediately prior to experimentation, routinely tested for mycoplasma (all negative) and showed loss of SMARCB1 as expected (Figure 1a). BT12, BT16 and CHLA266 were maintained in Iscove’s Modified Dulbecco’s Medium (IMDM) supplemented with 1% Insulin-Transferrin-Selenium, 20% foetal calf serum and 1% penicillin/streptomycin. CHLA04, CHLA05 and CHLA06 were maintained in KnockOut^TM^ DMEM/F012 supplement with FGF (20 ng/mL), EGF (20 ng/mL), 2% StemPro Neural Supplement and 1% penicillin/streptomycin. Human neural stem cells (NSC) were purchased from Life Technologies (Cat no. N7800–100) and maintained on fibronectin-coated plates in DMEM/F-12, FGF (20 ng/mL), EGF (20 ng/mL), 2% StemPro Neural Supplement and 1% penicillin/streptomycin. All cells were cultured in humidified 5% CO_2_/95% air atmosphere at 37 °C. Panobinostat (5 mmol/L; Selleckchem, Houston, United States of America) was prepared in 100% DMSO and stored in aliquots at −20 °C. Appropriate dilutions were made in culture medium at the time of experimentation.

### 2.2. Cell Viability

Cell viability was assessed using the alamarBlue^TM^ Cell Viability Reagent (ThermoFisher, Waltham, MA, USA) as instructed by the manufacturer. Cells were plated in 96 well plates at a density of 5 × 10^3^ cells/well and the following day, increasing concentrations of panobinostat (0.01–2000 nM) or DMSO vehicle control were added. Cells were treated for 72 h, with fresh treatments replenished every 24 h, and viability was determined. AlamarBlue was added to the medium and incubated at 37 °C for 4 h. Absorbance was measured with the CLARIOstar^®^ Plus High-Performance Monochromator Multimodal Microplate Reader (BMG LabTech, Cary, NC, USA) using a fluorescence excitation wavelength of 560 nm and an emission of 590 nm. Biological triplicate experiments were performed.

### 2.3. Analysis of Cell Cycle and Apoptosis by Flow Cytometry

A total of 2 × 10^4^ cells were plated in 6-well plates and the following day treated with panobinostat (10 nM and 100 nM) or DMSO control for 72 h with fresh treatments replenished daily. For analysis of cell cycle, cells were washed in PBS and fixed in 70% ethanol at −20 °C. Cells were then washed twice with PBS and stained using the FxCycle^TM^ PI/RNase Staining Solution (ThermoFisher, Waltham, MA, USA) as per manufacturers’ instructions. For analysis of cell death, cells were washed in PBS and resuspended in 1X Annexin V Binding Buffer (BD Pharmingen, Franklin Lakes, NJ, USA). FITC Annexin V (BD Pharmingen, Franklin Lakes, NJ, USA) and Propidium Iodide (BD Pharmingen, Franklin Lakes, NJ, USA) at a final concentration of 50 μg/mL was added. Flow cytometry was performed using the BD Biosciences FACSCanto II Analyzer and data was analysed using FlowJo Version 9.6.1 software (BD Bioscience, Franklin Lakes, NJ, USA).

### 2.4. Senescence-Associated β-Gal Staining and Clonogenic Assay

Cells (5 × 10^5^) were plated with complete culture media in 10-centimetre culture dishes, incubated overnight at 37 °C, and treated with 10 nM panobinostat or DMSO control for 21 days. Cell morphology was monitored throughout using the Incucyte^®^ Live Cell Analysis System (Essenbiosciences, Ann Arbor, MI, USA) with fresh treatments replenished every 24 h. Following 21 days culture, senescence-associated β-gal staining was performed as previously described [24]. For clonogenic assays, 5 × 10^3^ panobinostat and control-treated cells were plated in 6-well plates and cultured in normal growth medium for 14 days. After the culture period cells were washed in PBS, fixed in ice-cold methanol and stained with 0.005% crystal violet (Sigma Aldrich, St. Louis, MO, USA) for 30 min, before being extensively washed to remove residual stain and colonies >50 cells counted under an inverted microscope.

### 2.5. Global Gene Expression Analysis

BT12, BT16 and CHLA266 cells were collected from 10-centimetre culture dishes following 21 days culture in the presence of 10 nM panobinostat or DMSO. Biological triplicates were analysed. RNA was extracted from cell pellets using the RNeasy Mini Kit (Qiagen, Hilden, Germany) according to the manufacturer’s instructions, including an on-column DNase digestion step. RNA-seq analysis was performed by BGI (Hong Kong) to generate the FASTQ file. Read counts were generated from the FASTQ files by using RNA Structural Alignment Repository (RNA-STAR; Galaxy). The count matrix was used to obtain normalized reads as per Degust analysis package [26]. Primary gene expression data are available on the Gene Expression Omnibus (GEO) public database. The enrichment score for the cell pathways was further analysed using Gene Set Enrichment Analysis (GSEA) software as per manufacturer’s instruction [27,28]. Venn diagrams were constructed using Venn diagrams, an online open-source Venn diagram generator [29]. Gene Ontology Network analysis was performed using the ClueGO plugin of the open-source Cytoscape platform [30]. Cluego displayed a functionally related network based on the categorization of differentially expressed genes into gene ontology groups. Specific analysis of conserved gene ontology for biological processes was also performed via ClueGo.

### 2.6. Protein Analysis

A total of 5 × 10^5^ cells/well were plated in 6-well plates and the following day treated with 5, 10, 20, 50 and 100 nM panobinostat or DMSO control for 72 h. Cells were collected and western blot analysis was performed as previously described [24,31]. Membranes were probed with anti-rabbit BAF47 (SMARCB1) (BD Transduction Laboratories, no. 612110), anti-rabbit H3K27Ac (Cell Signaling Technology, no. 8173), anti-rabbit H3K27Me3 (Cell Signaling Technology, no. 9733), anti-rabbit Histone H3 (Cell Signaling Technology, no. 9715), anti-rabbit EZH2 (Cell Signaling Technology, no. 5246) and anti-mouse Actin (Abcam, ab11003), followed by donkey anti-mouse IRDye 680LT (Li-Cor Biosciences, no. 926-68022) and donkey anti-rabbit IRDye 800CW (Li-Cor Biosciences, no. 926-32213) secondary antibodies and visualized on the Odyssey CL-x System (LI-COR) as per the manufacturer’s instructions. Densitometric analysis of protein expression, normalized to Actin, was performed using the Odyssey CL-x System software.

### 2.7. Orthotopic ATRT Xenograft Model

BT12 cells expressing luc-GFP were generated following lentiviral transduction with pCMV Green firefly-luciferase plasmid (SystemBio; TR0XX) [32]. Transduced cells were validated with Xenolight D-Luciferin (Perkin Elmer; 122799). The validated cultures were then selected for GFP-expressing cells via cell sorting, renamed as BT12 luc-GFP, and expanded. A total of 5 × 10^5^ BT12 luc-GFP cells in 2ul PBS were engrafted into the cerebellum of NOD scid gamma (NSG) mice using a stereotaxic device (coordinates: x = 1, y = −1, z = −3 from lambda). To assess tumour growth, mice were imaged using the AMI-HTX bioluminescent imaging system (Spectral Instruments Imaging, Tucson, United States of America), 10 min following injection of 150 mg luciferin/kg body weight. Once tumour bioluminescence reached an intensity of 1 × 10^6^, relative light unit (RLU), mice were randomized to receive panobinostat (5 mg/kg) or vehicle control (5% dextrose) via intraperitoneal injection daily for 28 days or until ethical endpoints were reached. Ethical endpoints include neurological symptoms, >10% body weight loss or signs of general ill-health. At the completion of the study, mice were euthanized in a carbon dioxide chamber and whole brain was harvested for histology and immunohistochemical analysis. All experiments involving animals were approved in advance by an Animal Ethics Committee at Monash University and were carried out in accordance with “Australian Code of Practice for the Care and Use of Animals for Scientific Purposes”.

### 2.8. Histology and Immunohistochemistry

Formalin-fixed, paraffin-embedded tumour sections were analysed by histology after generating 4-micrometre tissue sections and staining with haematoxylin and eosin. Immunohistochemical stains were performed on representative tissue sections using H3K27Ac (Cell Signaling Technology, no. 8173), H3K27Me3 (Cell Signaling Technology, no. 9733), EZH2 (Cell Signaling Technology, no. 5246), BAF47 (SMARCB1) (BD Transduction Laboratories, no. 612110), Cleaved caspase-3 (Cell Signaling Technology, no. 9664S) and PCNA (Cell Signaling Technology, no. 2586S). Following sodium citrate (pH 6.0) antigen retrieval, staining was performed using the Vectastain ABC Elite Rabbit IgG Kit, ABC Elite Mouse IgG Kit or M.O.M^TM^ Kit (Vector Laboratories, Burlingame, United States of America) according to manufacturer’s instructions. Slides were counterstained with haematoxylin and imaged using the Nikon ECLIPSE TS100 bright-field microscope (Nikon, Minato City, Japan).

### 2.9. Statistical Analysis

All data were analysed with GraphPad Prism v9.0.2 (GraphPad Software, San Diego, CA, USA) and represented as mean ± SEM. For the statistical analysis test: An unpaired-two tailed T-test was used for 2 samples with a single variable; a one-way ANOVA followed by a Tukey’s multiple comparison test was used for more than 2 samples with 1 variable; a two-way ANOVA followed by a Sidak’s multiple comparison test was used to measure an interaction between 1 dependent variable and 2 independent variables; a log-rank (Mantel-Cox) test was used for contrast of Kaplan–Meier survival curves. A *p*-value of less than 0.05 was considered statistically significant and is symbolized by * <0.05, ** <0.01, *** <0.001, **** <0.0001. The number of samples (*n*) used for calculations is indicated in figure legends.

## 3. Results

### 3.1. Low-Dose Panobinostat Inhibits Human ATRT Cell Growth in the Absence of Cell Death

To evaluate the therapeutic efficacy of panobinostat in ATRT, we cultured a panel of human SMARCB1-deficient ATRT cell lines (BT12, BT16, CHLA04, CHLA05, CHLA06 and CHLA266) belonging to the ATRT-SHH and ATRT-MYC molecular subgroups and a neural stem cell (NSC) control cell line with intact SMARCB1 (Figure 1a, Supplementary Figure S7) in increasing concentrations of panobinostat. All ATRT cell lines demonstrated a dose-dependent reduction in cell viability with low nanomolar GI_50_ (range 20–60 nM) (Figure 1b, Supplementary Figure S1). Notably, the NSC cell line was far less responsive to panobinostat. To assess the on-target effects of panobinostat, acetylation of histone H3 at lysine 27 (H3K27Ac) was evaluated by western blot analysis following 72 h of continuous treatment. A significant increase in H3K27Ac was observed exclusively in SMARCB1 null ATRT (BT12, BT16, CHLA266) with increasing concentrations of panobinostat (Figure 1c, Supplementary Figures S2 and S7). Conversely, trimethylation of Histone H3 lysine 27 (H3K27Me3) and expression of EZH2, a member of the Polycomb Repressive Complex 2 (PRC2) that catalyses H3K27Me3, were progressively reduced with increasing concentrations of panobinostat in SMARCB1 null ATRT (Figure 1c, Supplementary Figures S2 and S7). An increase in H3K27Ac and decrease in H3K27Me3 was detected after treatment with as little as 5–20 nM panobinostat and was maximally affected at concentrations of 50–100 nM. In contrast, little-to-no change in H3K27Ac, H3K27Me3 or EZH2 was detected in the NSC cell line (Figure 1c, Supplementary Figures S2 and S7).

Cell cycle analysis following 72 h of sustained treatment with DMSO control, low-dose panobinostat (10 nM) or high-dose panobinostat (100 nM), concentrations either side of the GI_50_, demonstrated a significant accumulation of cells in G0-G1 (DMSO, 65.5% ± 0.9% vs. 10 nM panobinostat, 68.4% ± 0.9% and 100 nM panobinostat, 73.8% ± 0.9%) and a reduced proportion of cells in S-phase (DMSO, 14.8% ± 0.7% vs. 10 nM panobinostat, 7.1% ± 0.7% and 100 nM panobinostat, 6.4% ± 0.8%) in BT12 ATRT cells (Figure 1d). A marked increase in G2-M was also observed with 10 nM panobinostat. Similar results were also observed in BT16 (3.0% and 8.0% increase in G0-G1 and 7.0% and 7.1% decrease in S-phase for 10 nM and 100 nM panobinostat, respectively) and CHLA266 (3.3% and 5.0% increase in G0-G1 and 8.4% and 7.9% decrease in S-phase for 10 nM and 100 nM panobinostat, respectively) (Supplementary Figure S3). Notably, analysis of apoptosis by Annexin V staining revealed that low-dose panobinostat (10 nM) does not cause cell death in BT12, BT16 and CHLA266 ATRT cells, suggesting it is not cytotoxic at this concentration (Figure 1e, Supplementary Figure S3). However, high-dose panobinostat (100 nM) did elicit cell death, resulting in increased proportions of apoptotic cells compared to low-dose panobinostat or vehicle control (BT12, 76.6% vs. 0.1% and 0.1%; BT16 38.3% vs. 0.1% and 0.1%; CHLA266 79.6% vs. 0.1% and 0.1%) (Figure 1e, Supplementary Figure S3). This is consistent with our morphological observations (Supplementary Figure S1).

### 3.2. Continuous Low-Dose Panobinostat Treatment Inhibits Cell Growth, Clonogenecity and Induces Cellular Senescence in Human ATRT Cells

**Figure 2:**
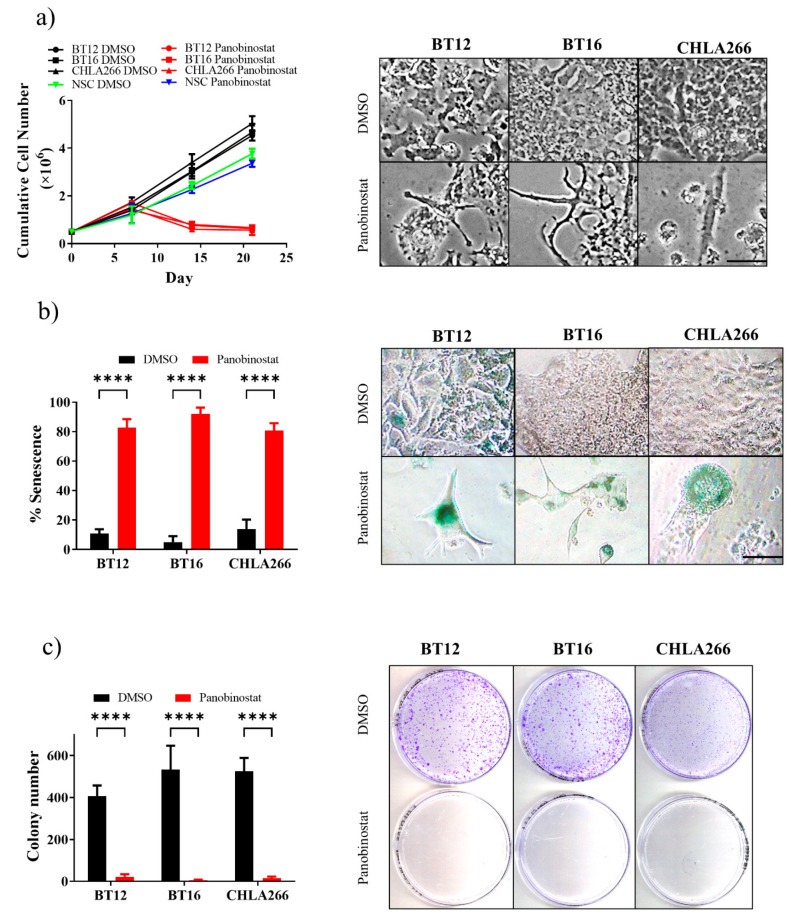
Continuous 10 nM panobinostat treatment reduces cell growth, colony formation and induces cellular senescence in human ATRT cells. (**a**) Cumulative cell growth of ATRT cells, BT12, BT16, and CHLA266, and NSC cells challenged with continuous 10 nM panobinostat or DMSO vehicle control for 21 days (*n* = 3; mean ± SEM). Light microscopy images depict cellular morphology of BT12, BT16, and CHLA266 following 21 days treatment. Scale bar = 100 µm. (**b**) Quantitation and light microscopy of senescence-associated β-galactosidase assay on ATRT cells, BT12, BT16, and CHLA266, treated with sustained 10 nM panobinostat or DMSO vehicle control for 21 days (*n* = 3; mean ± SEM; ****, *p* < 0.0001). Scale bar = 100 µm. (**c**) Quantitation and representative images of colony formation potential in ATRT cells, BT12, BT16, and CHLA266, treated with continuous 10 nM panobinostat of DMSO vehicle control for 21 days (*n* = 3; mean ± SEM; ****, *p* < 0.0001).

Since low-dose panobinostat increases histone acetylation and promotes cell growth arrest in the absence of cell death following short-term 72 h exposure, we explored the effects of prolonged low-dose panobinostat treatment. BT12, BT16 and CHLA266 cells cultured in the presence of 10 nM panobinostat for 21 days exhibited a marked reduction in cumulative cell number compared to the DMSO vehicle-control treatments (Figure 2a). Consistent with the 72-h cultures, no difference in cell growth was detected between panobinostat and vehicle-control NSC cultures (Figure 2a). Cell growth arrest after approximately seven days of sustained treatment was accompanied by progressive changes in cell morphology, characterized by a flattened phenotype and the presence of cellular projections (Figure 2a). The flattened cell phenotype and G0–G1 cell-cycle arrest was consistent with cell senescence. Indeed, senescence-associated β-galactosidase staining was significantly increased following sustained low-dose panobinostat treatment in BT12 (DMSO, 10.7 ± 1.8 vs. 10 nM panobinostat, 82.7 ± 3.4; *p* < 0.0001), BT16 (DMSO, 5.0 ± 2.3 vs. 10 nM panobinostat, 92.0 ± 2.5; *p* < 0.0001) and CHLA266 (DMSO, 13.7 ± 3.8 vs. 10 nM panobinostat, 80.7 ± 3.0; *p* < 0.0001) compared to their vehicle controls (Figure 2b). Furthermore, the clonogenicity of human ATRT cells treated with continuous low-dose panobinostat for 21 days was almost completely ablated (Figure 2c). Colony formation was significantly reduced in BT12 (DMSO, 406.7 ± 29.5 vs. 10 nM panobinostat, 22.3 ± 6.7; *p* < 0.0001), BT16 (DMSO, 533.7 ± 65.7 vs. 10 nM panobinostat, 4.7 ± 2.0; *p* < 0.0001) and CHLA266 (DMSO, 524.7 ± 37.2 vs. 10 nM panobinostat, 14.7 ± 4.9; *p* < 0.0001), respectively. Collectively, these findings demonstrate that continuous low-dose panobinostat potently inhibits human ATRT cell growth and clonogenicity.

### 3.3. Gene Expression Profiles Characteristic of Growth Arrest and Nervous System Development Are Induced by Sustained Low-Dose Panobinostat Treatment in Human ATRT Cells

**Figure 3:**
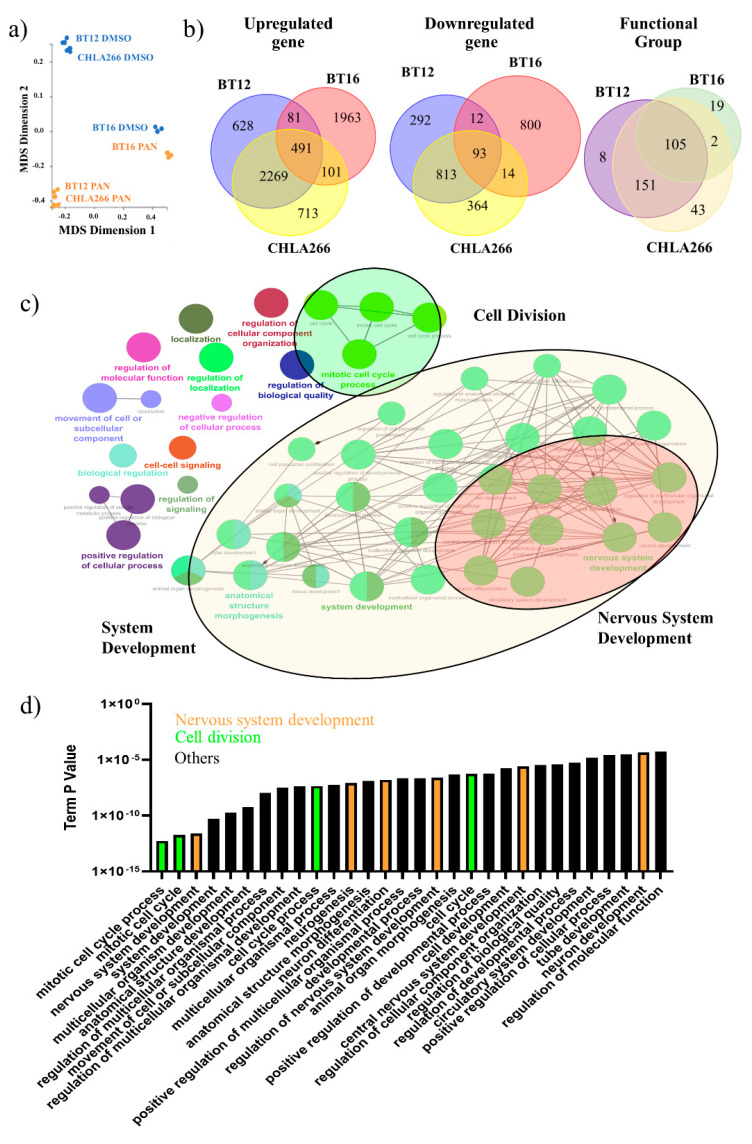
Global gene expression analysis of human ATRT cell lines treated with panobinostat. (**a**) Multidimensional scaling (MDS) plot of BT12, BT16 and CHLA266 treated with DMSO or 10 nM panobinostat (PAN) for 21 days (*n* = 3). (**b**) Venn diagram of differentially expressed genes and functional groups in BT12, BT16, and CHLA266 treated with 10 nM panobinostat (*n* = 3). (**c**) Gene Ontology network map for conserved differentially expressed genes among BT12, BT16 and CHLA266 treated with 10 nM panobinostat for 21 days (FDR < 0.05; LogFc < −2 or >2; pV < 0.05). (**d**) Most significant functional groups associated with conserved differentially expressed genes among BT12, BT16 and CHLA266 treated with 10 nM panobinostat for 21 days.

To define gene expression changes following low-dose panobinostat treatment we performed genome-wide transcriptional profiling by RNA-seq on BT12, BT16 and CHLA266 following 21 days of treatment. Multidimensional scaling of the RNA-seq data showed a complete overlap of triplicate samples and a distinct separation between vehicle and panobinostat treatments for each cell line (Figure 3a). Hierarchical cluster analysis identified 4681 (3470 upregulated, 1211 downregulated), 3557 (2637 upregulated, 920 downregulated) and 4860 (3575 upregulated, 1,285 downregulated) differentially expressed genes in BT12, BT16 and CHLA266 respectively (Figure 3b). Of these, 584 genes (491 upregulated, 93 downregulated) were conserved between BT12, BT16 and CHLA266 (Figure 3b).

**Figure 4:**
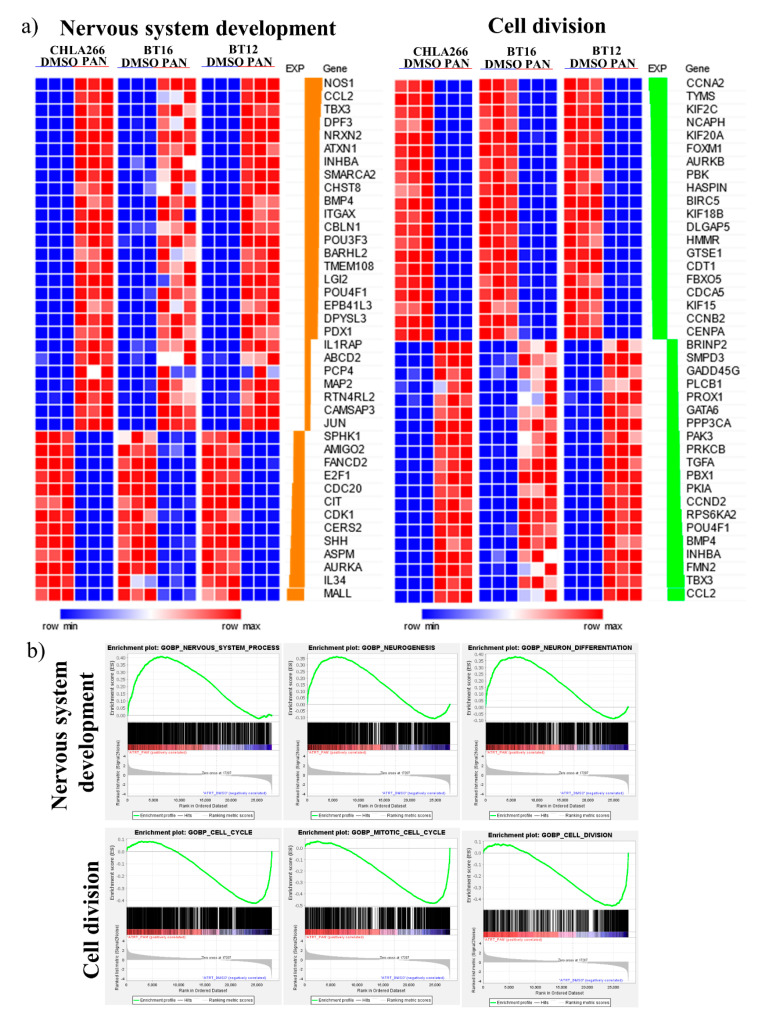
Sustained panobinostat treatment of ATRT cells regulates genes associated with nervous system development and cell division. (**a**) Heatmap representation of differentially expressed genes associated with nervous system development (left) and cell division processes (right). Each column represents a distinct sample and each row, a distinct gene. Level of expression is denoted by colour (blue, low; red, high). (**b**) GSEA enrichment plot of functional groups involved in nervous system development (top) and cell division processes (bottom).

Independent gene ontology analysis of differentially expressed genes for each cell line revealed the enrichment of functional groups associated with embryonic development, in particular nervous system development and differentiation (Supplementary Figures S4–S6). Furthermore, gene ontology analysis of the 584 conserved differentially expressed genes across all cell lines revealed an overlap of 105 functional groups associated with development and differentiation, including nervous system development, forebrain development, spinal cord development, cell differentiation, neurogenesis, neuron differentiation, tissue development, tissue morphogenesis and embryonic morphogenesis (Figure 3c,d). Notably, functional groups associated with nervous system development were most frequently observed. Detailed inspection of these functional groups further revealed enrichment of genes that are essential in nervous system development (Figure 4a,b). Several key promoters and regulators in central nervous system development (Nitric Oxide Synthase 1 (NOS1) [33,34,35,36]), neuron differentiation (T-box Transcription Factor 3 (TBX3) [37], Desmoplakin 3 (DPF3) [38,39], Bone Morphogenetic Protein 4 (BMP4) [37,40]) and neuronal development (Neurexin 2 (NRXN2) [41,42], Ataxin 1 (ATXN1) [43,44]) were upregulated, supporting neuronal differentiation [45] (Figure 4a,b). Additionally, there was also a prevalence of functional groups associated with cell division regulation including the mitotic cell cycle, nuclear division, regulation of metaphase/anaphase transition of the cell cycle, regulation of chromosome separation and spindle organization (Figure 3c,d). Detailed inspection of these functional groups further identified differential expression of critical genes associated with cell division, including Cyclin A2 (CCNA2) [46,47,48], that regulates the G1-M cell-cycle transitions, Histone H3 Associated Protein Kinase (HASPIN), that controls the metaphase of the cell cycle, Survivin (BIRC5) [48,49], Foxhead Box Protein M1 (FOXM1) [50,51], Aurora Kinase B (AURKB) [52,53], PDZ Binding Kinase (PBK) [54], that regulates the mitosis phase of the cell cycle, and Kinesin Family Member 20a (KIF20A) [55,56] and Kinesin Family Member 18B (KIF18B) [57,58], that modulate cytokinesis during mitosis (Figure 4a,b). These genes are important in regulating the cell cycle and their downregulation is consistent with the observed growth arrest phenotype. Collectively, these data support the growth inhibitory effects of sustained low-dose panobinostat treatment in human ATRT cell lines and strongly implicates the induction of large sets of genes required for normal nervous system development and neuronal differentiation.

### 3.4. Sustained Panobinostat Treatment inhibits Tumour Growth and Extends Survival in an Orthotopic In Vivo ATRT Xenograft Model

**Figure 5:**
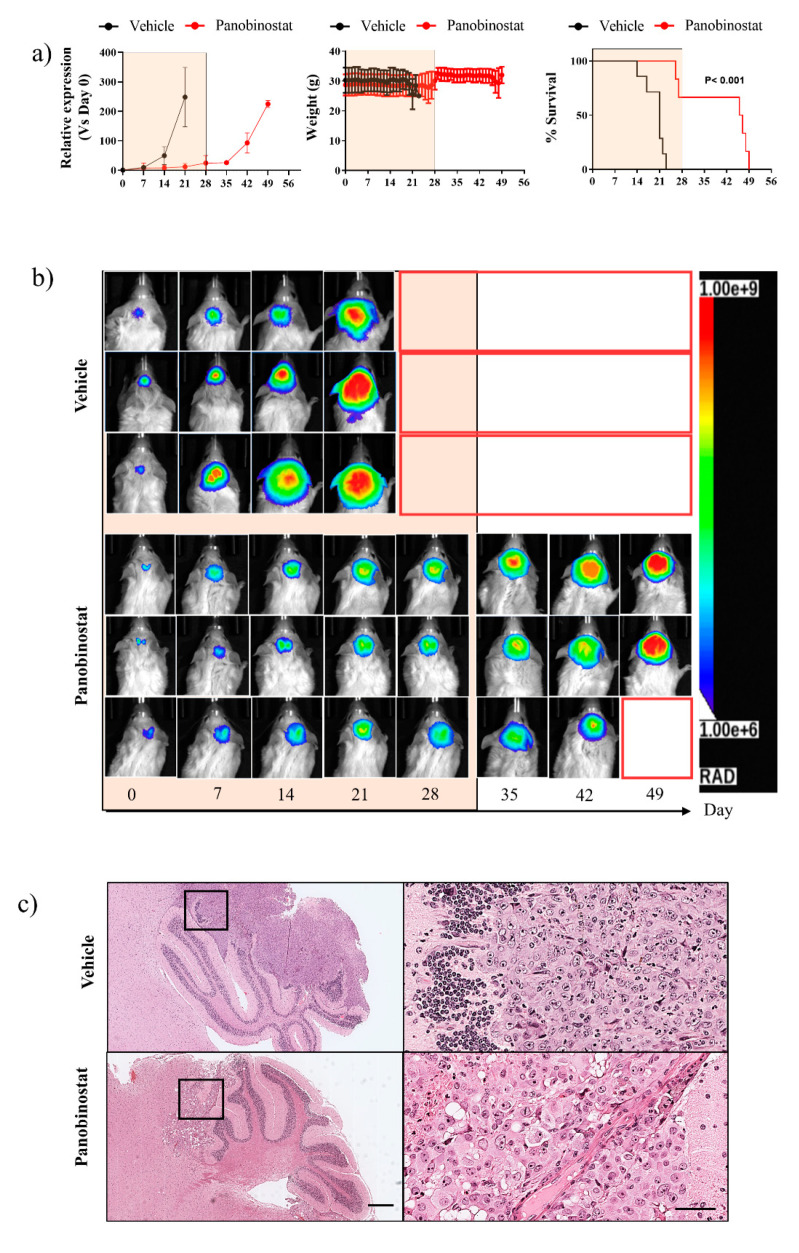
Orthotopic ATRT xenograft model treated with vehicle control or 5 mg/kg panobinostat daily for 28 days. (**a**) Relative bioluminescence intensity of engrafted BT12 Luc-GFP tumours, mouse body weight and the survival proportion of mice (*n* = 7; mean ± SEM, *p* < 0.001). Orange box represents the treatment period. (**b**) Representative bioluminescence images of NSG mice engrafted with BT12 Luc-GFP and treated with vehicle control or 5 mg/kg panobinostat daily for 28 days. Orange box on *X*-axis represents the treatment period. Treatment withdrawal occurred on Day 28. (**c**) Representative H&E image of brains harvested from mice following treatment with vehicle control or 5 mg/kg panobinostat daily for 28 days. Scale bar for left image = 500 µm; right image = 200 µm.

To better evaluate the effect of low-dose panobinostat, we generated a luciferase-GFP-tagged BT12 orthotopic intracranial xenograft mouse model. Following intracranial injection of 5 × 10^5^ cells into the cerebellum of NSG mice, engraftment, visualised by bioluminescence, was observed in 70% of mice within three weeks of surgery, and 90% within five weeks of surgery. Upon reaching a bioluminescence intensity of 1 × 10^6^, mice were randomly assigned to receive vehicle control or 5 mg/kg panobinostat daily via intraperitoneal injection. We previously demonstrated that this dose is tolerable and efficacious in both osteosarcoma and extracranial malignant rhabdoid tumour models [23,24]. Vehicle control-treated mice demonstrated progressive tumour growth and a median survival of 21 days (range 14–23 days) following commencement of treatment (Figure 5a,b). In contrast, tumours in panobinostat-treated mice showed reduced growth and, in most cases, underwent complete growth arrest approximately seven days after the initiation of treatment. However, following the withdrawal of panobinostat treatment after 28 days, tumour progression ultimately occurred, and mice reached ethical endpoints approximately 14 days later with a median survival of 46 days (range 26–49 days, *p* < 0.001) (Figure 5b). It is worth noting the two panobinostat-treated mice that succumbed during treatment had more advanced disease, bioluminescence intensities of 5 × 10^6^ and 7 × 10^6^, at the commencement of treatment, suggesting that lower disease burden is associated with increased panobinostat effectiveness. Consistent with previous studies, no systemic effects of panobinostat treatment were observed, suggesting it is well tolerated in this orthotopic model (Figure 5a). These findings demonstrate the efficacy of sustained low-dose panobinostat treatment in ATRT in the absence of systemic toxicity.

**Figure 6:**
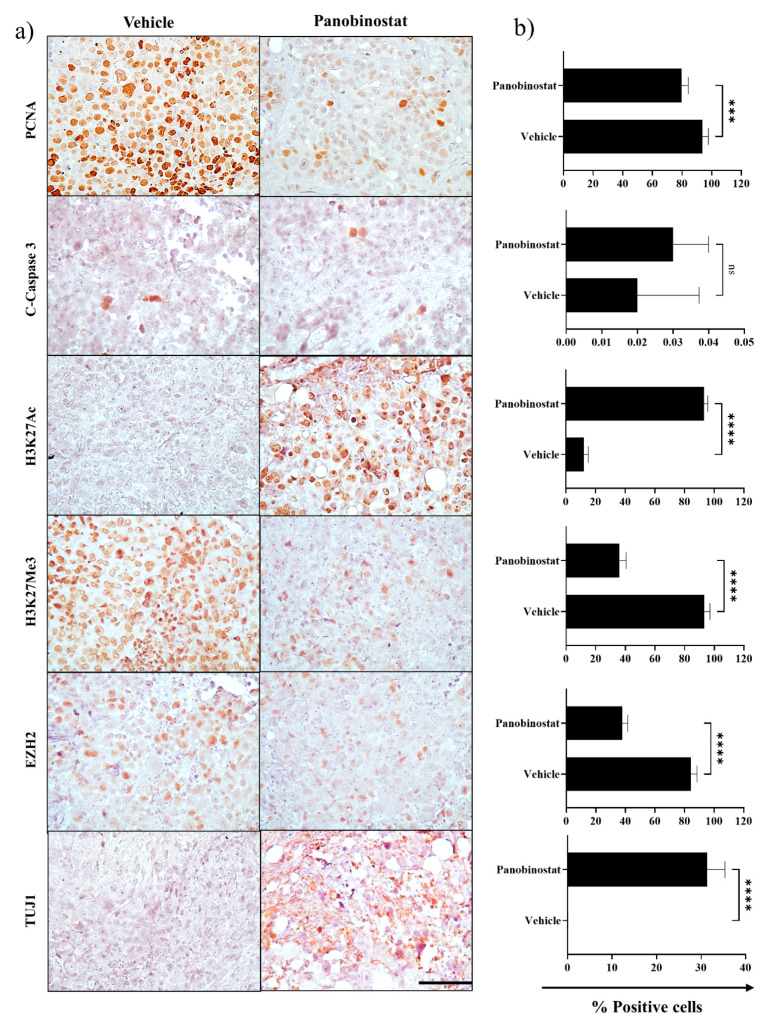
Immunohistochemical analysis of tumours from the BT12 Luc-GFP orthotopic xenograft model following sustained treatment with panobinostat or vehicle control. (**a**) Immunohistochemical analysis for PCNA, C-Caspase 3, H3K27Ac, H3K27Me3, EZH2 and TUJ1. Scale bar = 200 µm. (**b**) Quantitation of immunohistochemical staining (*n* = 7; mean ± SEM; ****, *p* < 0.0001).

Histopathological analysis of tumours from a cohort of mice collected at the completion of 28 days treatment or ethical endpoint, whichever came first, revealed a significantly lower tumour burden in the panobinostat-treated mice compared to vehicle controls, consistent with bioluminescent imaging (Figure 5c). Vehicle and panobinostat treated tumours revealed typical rhabdoid features, including well-defined cell borders, abundant cytoplasm with eosinophilic inclusions and eccentrically located nuclei. Immunohistochemical analysis of panobinostat-treated tumour tissue revealed a significant reduction in cell proliferation compared to vehicle control, as assessed by PCNA staining (vehicle, 93.7 ± 5.1 vs. panobinostat, 79.6 ± 4.5; *p* < 0.0001 (Figure 6a,b). No change in cleaved caspase-3 staining, a measure of cell death, was detected, consistent with a non-cytotoxic effect of low-dose panobinostat (vehicle, 0.02 ± 0.01 vs. panobinostat, 0.03 ± 0.02; *p* > 0.999). In addition, sustained low-dose panobinostat treatment in vivo led to a significant increase in H3K27Ac (vehicle, 12.0 ± 3.1 vs. panobinostat, 93.0 ± 2.6; *p* < 0.0001) and decrease in H3K27Me3 (vehicle, 93.3 ± 3.8 vs. panobinostat, 36.0 ± 4.6 vs. *p* < 0.0001) and EZH2 (vehicle, 84.3 ± 4.1 vs. panobinostat, 38.0 ± 3.6; *p* < 0.0001). Furthermore, panobinostat-treated mice also demonstrated a significant focal increase in neuron-specific class 3 β-tubulin (TUJ1) (vehicle, 0.05 ± 0.03 vs. panobinostat, 31.3 ± 4.1; *p* < 0.0001), a neuronal marker, implicating neuronal differentiation (Figure 6). Collectively, these findings demonstrate the efficacy of low-dose panobinostat as a differentiation therapy for ATRT.

## 4. Discussion

ATRT is an aggressive malignancy primarily affecting infants and young children with poor outcomes, highlighting the urgent need for improved therapeutic strategies. As an emerging class of anticancer therapy, HDACi has been explored in a large range of hematological and solid tumours [23,24,59]. Here, we show that sustained, low-dose panobinostat treatment inhibits tumour growth and drives neuronal differentiation in ATRT.

### 4.1. ATRT Is Amenable to Differentiation Therapy

ATRT is characterized by an undifferentiated phenotype with a heterogenous histopathology consisting of varying amounts of progenitor neuronal ectoderm or mesenchymal elements [60,61,62] and expression of embryonic stem cell markers including Sal-like Protein 4 (SALL4), Lin-28 Homolog A (LIN28A), Glypican 3, SALL4, T-cell leukaemia 1 (TCL1) and Undifferentiated embryonic cell Transcription Factor 1 (UTF1) [16,63]. These observations have led to speculation that ATRT may arise from pluripotent embryonic stem cells. Furthermore, recent next-generation sequencing studies demonstrate an overlap between SMARCB1-dependent promoter targets and tissue-specific lineage-determining genes that are downregulated in SMARCB1-deficient malignant rhabdoid tumours [62]. Notably, germ cell factors including Placenta Alkaline Phosphatase (PLAP), Alkaline feto-protein (AFP), Octamer-binding transcription factor 4 (OCT4), Receptor Tyrosine Kinase (c-Kit) and β−human chorionic gonadotrophin (β-HCG) are not expressed, suggesting incomplete lineage differentiation of ATRT [64]. Of particular relevance to this study, the inactivation of *SMARCB1* in human pluripotent stem cells impairs neuronal differentiation and maintains the cells in an undifferentiated state [19]. This is consistent with our data in ATRT showing the upregulation of large sets of genes associated with neuronal-lineage development and differentiation, including nervous system development, forebrain development, hindbrain development, spinal cord development, cell differentiation, neurogenesis and neuron differentiation, following sustained low-dose panobinostat treatment. Furthermore, increased expression of the neuronal marker, TUJ1, in tumour tissue from an orthoptopic ATRT xenograft model treated with panobinostat further supports the potential for ATRT to undergo neuronal differentiation. Concurrently, we also demonstrate a downregulation of key regulators involved in cell division in response to sustained panobinostat treatment. This result is further supported by decreased PCNA expression, a marker for proliferation and cell division, in tumour tissue from an orthoptopic ATRT xenograft model treated with panobinostat and further demonstrates the potential of low-dose panobinostat to inhibit cell growth.

### 4.2. Efficacy of Sustained Low-Dose Panobinostat in Attenuating ATRT Growth

Panobinostat is a pan-HDACi that is FDA- and TGA-approved for the treatment of refractory multiple myeloma [65] and has been extensively studied in a range of other hematological and solid tumours. However, its application to brain tumours is somewhat controversial due to concerns about its ability to permeate the blood-brain barrier (BBB), rapid clearance from the tumour bed and is a substrate of P-glycoprotein, the BBB’s drug efflux transporter [66,67,68,69,70]. Moreover, a recent murine study in an orthotopic DIPG xenograft model showed significant toxicity associated with systemic administration of panobinostat up to 20 mg/kg/day, including diarrhea, myelosuppression and arrhythmias [71]. In this study, we demonstrate the efficacy and on-target activity of sustained low-dose panobinostat (5 mg/kg/day; intraperitoneal route) in inducing tumour growth arrest and improving overall survival in the absence of systemic toxicity, suggesting that therapeutically beneficial concentrations of panobinostat can reach the tumour in our orthotopic model. Importantly, Homan et al. have recently shown that panobinostat concentrations can accumulate in the murine brain at concentrations above the IC-50 described for DIPG and propose that panobinostat is effective for CNS tumours where the IC-50 is in the low nanomolar range, supporting its utility for ATRT [72]. However, further PK/PD studies are required to determine the CNS concentrations of panobinostat following low-dose chronic exposure. It should also be acknowledged that murine orthotopic models may not truly reflect the human BBB and as a result, outcomes from trials will be important. As such, the outcomes from an ongoing Phase II study of continuous, low-dose panobinostat in paediatric ATRT is eagerly awaited (ACTRN12618000321246). To ensure sufficient delivery of panobinostat to the tumour bed, studies are currently underway to determine the efficacy of convention-enhanced delivery (CED) of MTX110, a solubilised nanoparticle formulation of panobinostat, in CNS tumours including DIPG (NCT03566199). Whether CED is amenable to the chronic administration of treatments will need to be determined. Nevertheless, understanding the precise mechanisms of HDACi-mediated differentiation in ATRT will be critical in enabling the modification and/or development of BBB penetrant drugs.

The response to panobinostat was maintained during active treatment, with mice demonstrating tumour growth arrest. However, increasing bioluminescence intensities were observed in panobinostat-treated mice in the first seven days of therapy before stabilising. Albeit, any tumour progression during early panobinostat treatment was markedly less than in vehicle controls, suggesting panobinostat does have some immediate effect and is maximal after a sustained period. Notably, tumour progression did occur after the withdrawal of panobinostat, suggesting a heterogenous tumour response to treatment. This is consistent with a previous study in which tumour recurrence was observed at the completion of a three-week panobinostat treatment in a transgenic neuroblastoma model [73]. Remarkably, an extended panobinostat treatment period of nine weeks in the neuroblastoma model resulted in complete terminal differentiation of the tumour and was curative [73]. We show focal TUJ1 expression in the BT12 orthotopic xenograft model treated with panobinostat for 28 days, suggesting that a longer duration of treatment is likely required to drive more extensive neuronal differentiation and a more durable tumour response. Although ATRT-MYC subgroup models were predominantly used in this study, we also show that the ATRT-SHH subgroup cell lines, CHLA04 and CHLA05, are equivalently responsive to panobinostat (Figure 1b). This is consistent with a report showing significant growth inhibition of ATRT cell lines with the pan-HDACi dacinostat (LQ824), irrespective of molecular subtype [74]. Furthermore, the overexpression of HDAC1/2 across all ATRT subgroups [14] strongly supports the broad application of HDACi-mediated differentiation therapy in ATRT.

## 5. Conclusions

We demonstrated the efficacy of sustained low-dose panobinostat to inhibit ATRT tumour growth and induce neuronal differentiation, highlighting the therapeutic potential of epigenetic differentiation therapy to treat this disease.

## Data Availability

Primary gene expression data are available on the Gene Expression Omnibus (GEO) public database.

## Supplementary Materials

The following are available online at https://www.mdpi.com/article/10.3390/cancers13205145/s1, Figure S1: Representative images of NSC, BT12, BT16, and CHLA266 human ATRT cells treated with DMSO or 5 to 100 nM panobinostat for 72 h, Figure S2: Densitometry analysis of EZH2, H3K27Me3 and H3K27Ac in BT12, BT16, and CHLA266 and NSC cells after treatment with increasing doses of panobinostat (5 nM–100 nM) for 72 h, Figure S3: Cell cycle analysis of BT16, and CHLA266 treated with increasing doses of panobinostat (10–100 nM) for 72 h, Figure S4: Gene ontology network map for differentially expressed genes in BT12 human ATRT cells treated with 10 nM panobinostat for 21 days, Figure S5: Gene ontology network map for differentially expressed genes in BT16 human ATRT cells treated with 10 nM panobinostat for 21 days, Figure S6: Gene ontology network map for differentially expressed genes in CHLA266 human ATRT cells treated with 10 nM panobinostat for 21 days, Figure S7: Full western blot images including densitometry values.
