# Supplementary material for: Atypical Teratoid Rhabdoid Tumours Are Susceptible to Panobinostat-Mediated Differentiation Therapy
Source: Cancers (Basel). 2021 Oct 14;13(20):5145. doi: 10.3390/cancers13205145 (PMC8534272; doi:10.3390/cancers13205145)
Supplement: Supplementary file 1 [file cancers-13-05145-s001.zip › cancers-1379707-supplementary.pdf]

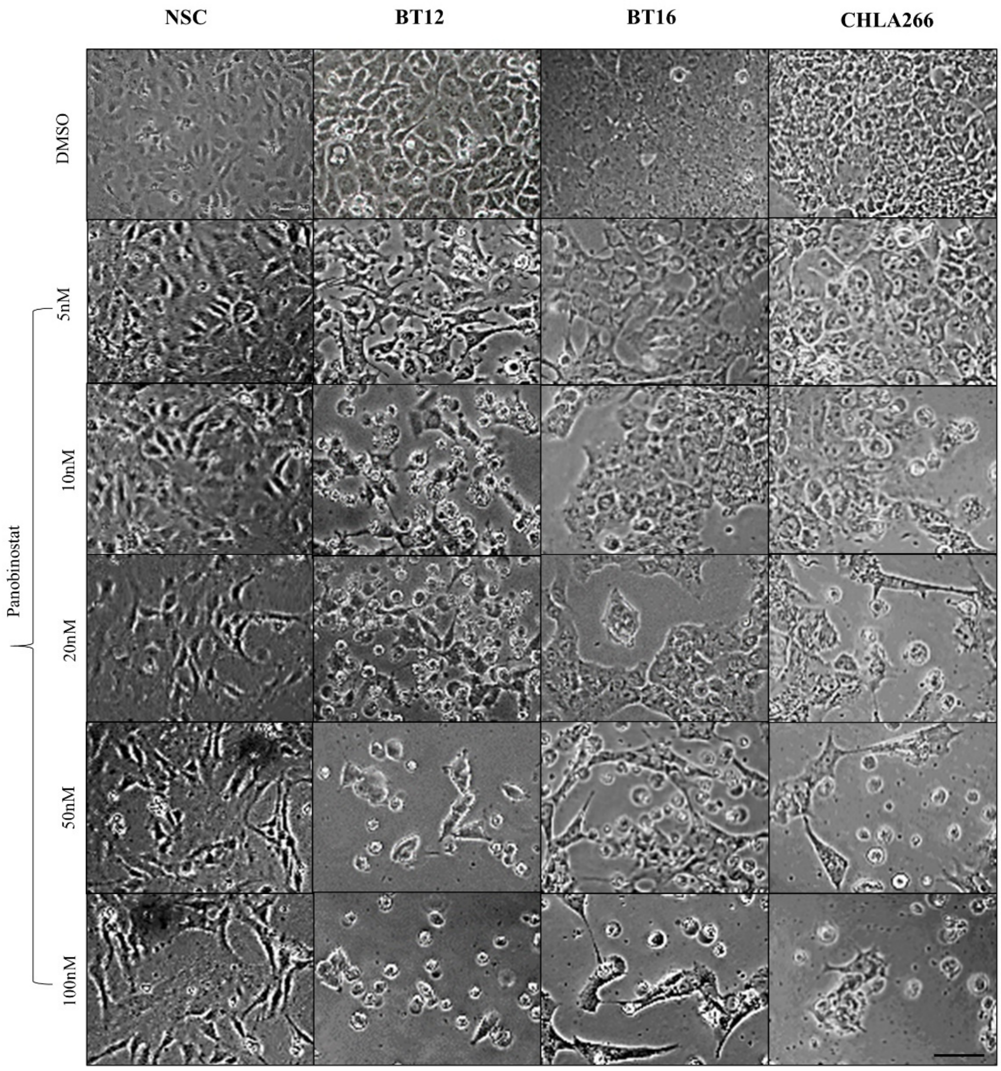

Supplementary Figure S1: Representative images of NSC, BT12, BT16, and CHLA266 human ATRT cells treated with DMSO or 5 to 100nM panobinostat for 72 hours. Scale bar = 100μm.

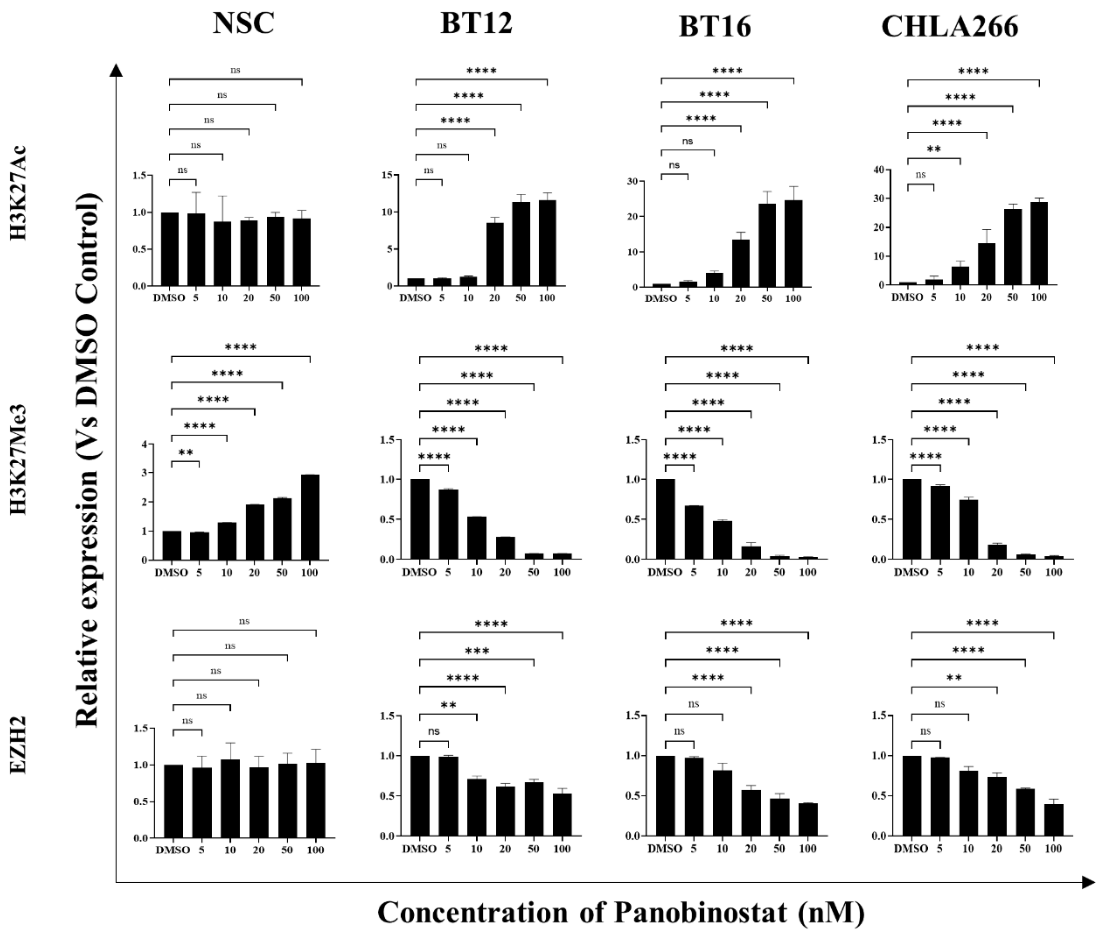

Supplementary Figure S2: Densitometry analysis of EZH2, H3K27Me3 and H3K27Ac in BT12, BT16, and CHLA266 and NSC cells after treatment with increasing doses of panobinostat (5nM – 100nM) for 72 hours. (*n*=3; mean ± SEM; ns, not significant; *, p <0.05; **, p <0.01; ***, p <0.001; ****, p <0.0001).

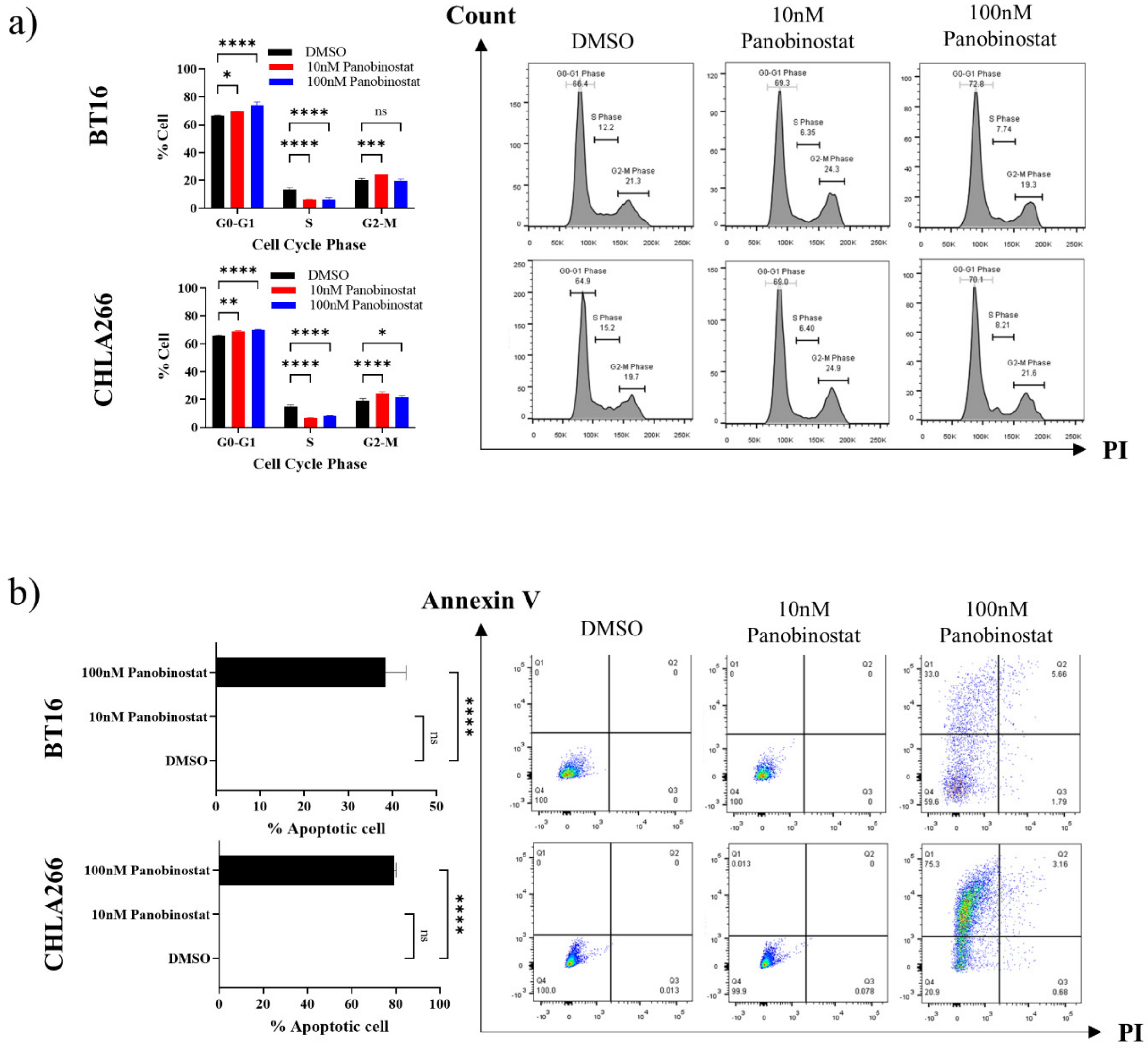

Supplementary Figure S3. (**a**) Cell cycle analysis of BT16, and CHLA266 treated with increasing doses of panobinostat (10–100nM) for 72 hours (n=3; mean ± SEM; ns, not significant; *, p <0.05; **, p <0.01; ***, p <0.001; ****, p <0.0001). (**b**) Annexin V assay of BT16, and CHLA266 treated with increasing doses of panobinostat (10–100nM) for 72 hours (n=3; mean ± SEM; ns, not significant; ****, p <0.0001).

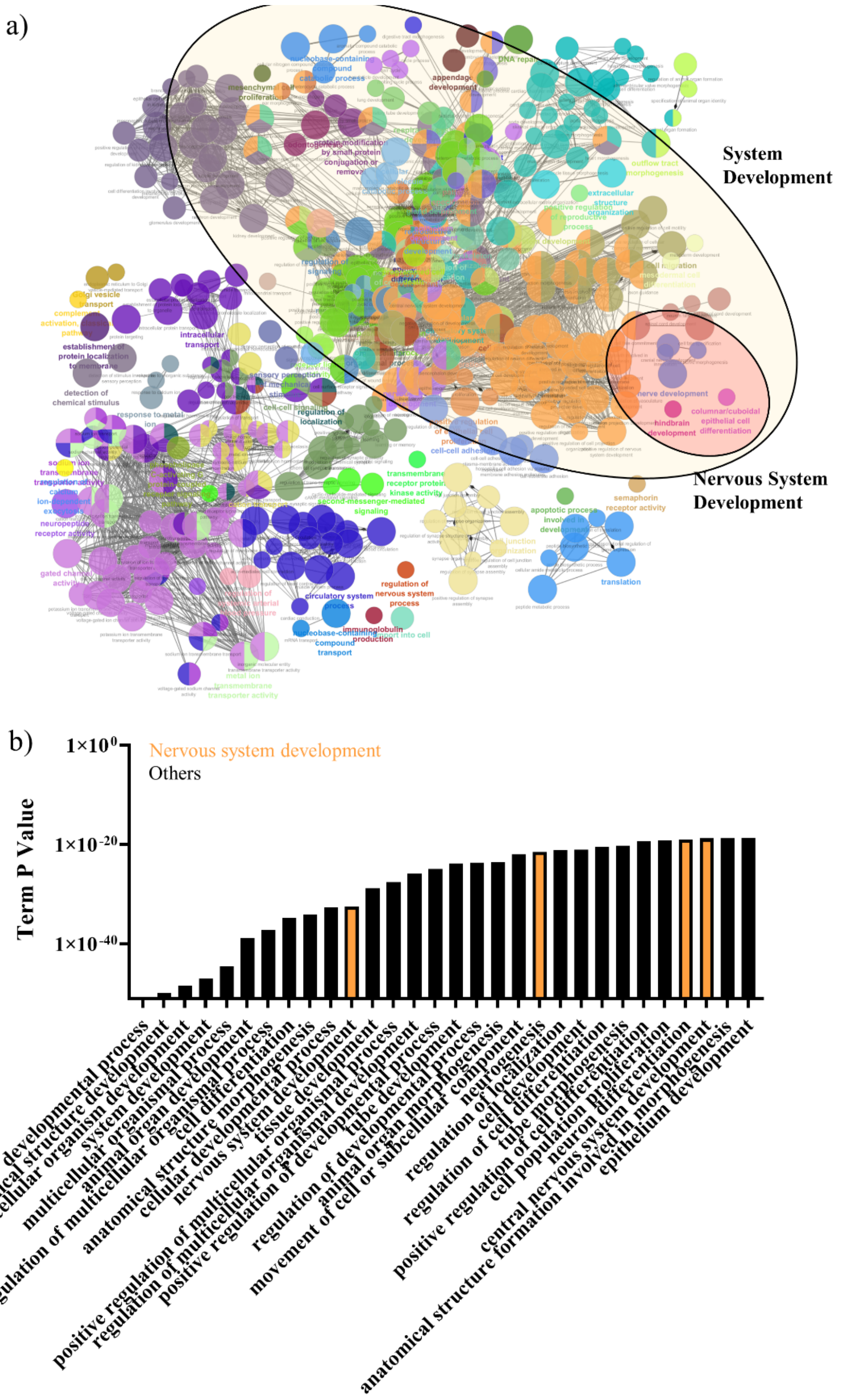

Supplementary Figure S4: (**a**) Gene ontology network map for differentially expressed genes in BT12 human ATRT cells treated with 10nM panobinostat for 21 days (FDR< 0.05; LogFc<-2 or >2; pV<0.05). (**b**) Most significant functional groups associated with differentially expressed genes.

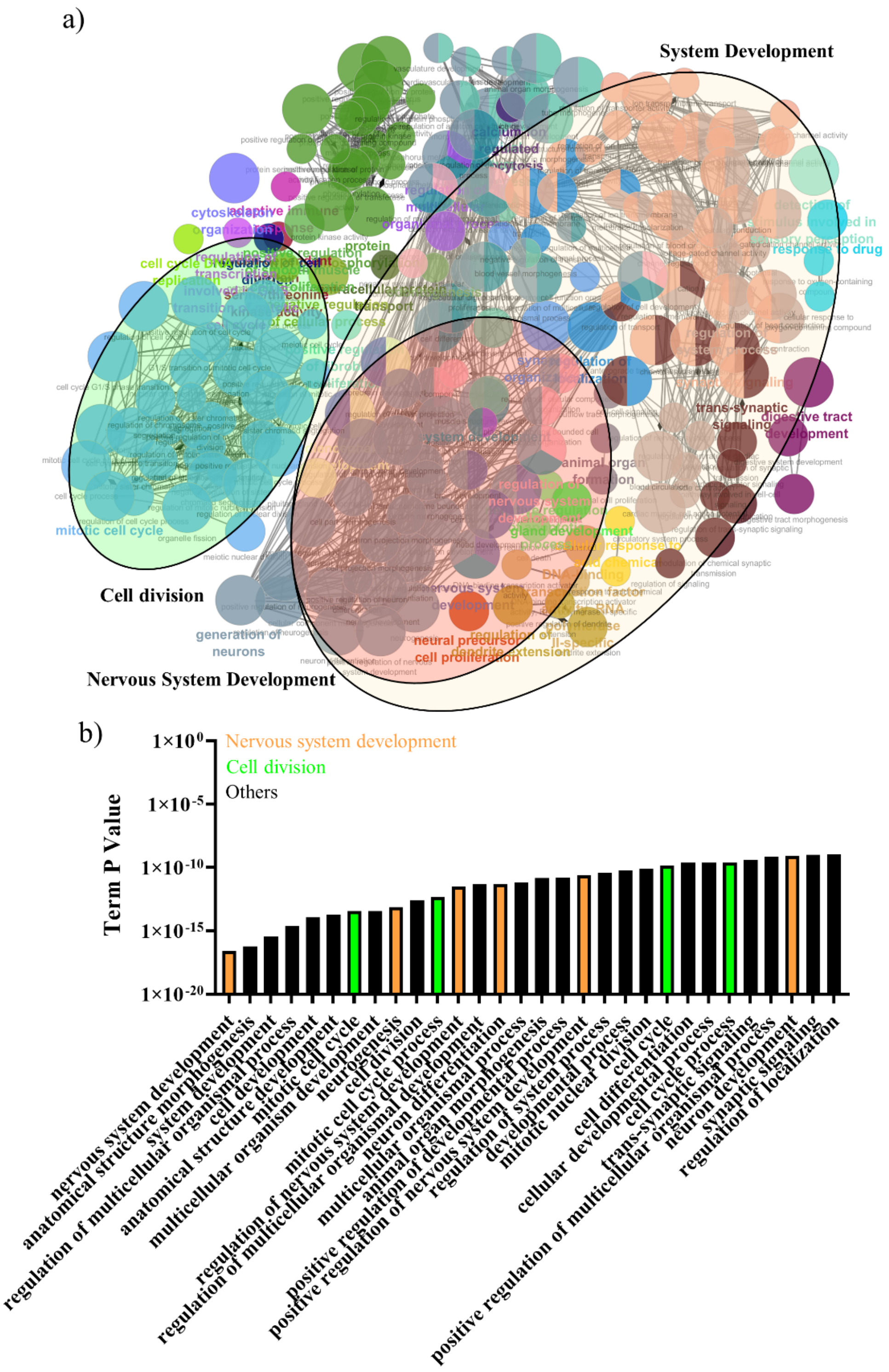

Supplementary Figure S5: (**a**) Gene ontology network map for differentially expressed genes in BT16 human ATRT cells treated with 10nM panobinostat for 21 days (FDR< 0.05; LogFc<-2 or >2; pV<0.05). (**b**) Most significant functional groups associated with differentially expressed genes.

a)

b)

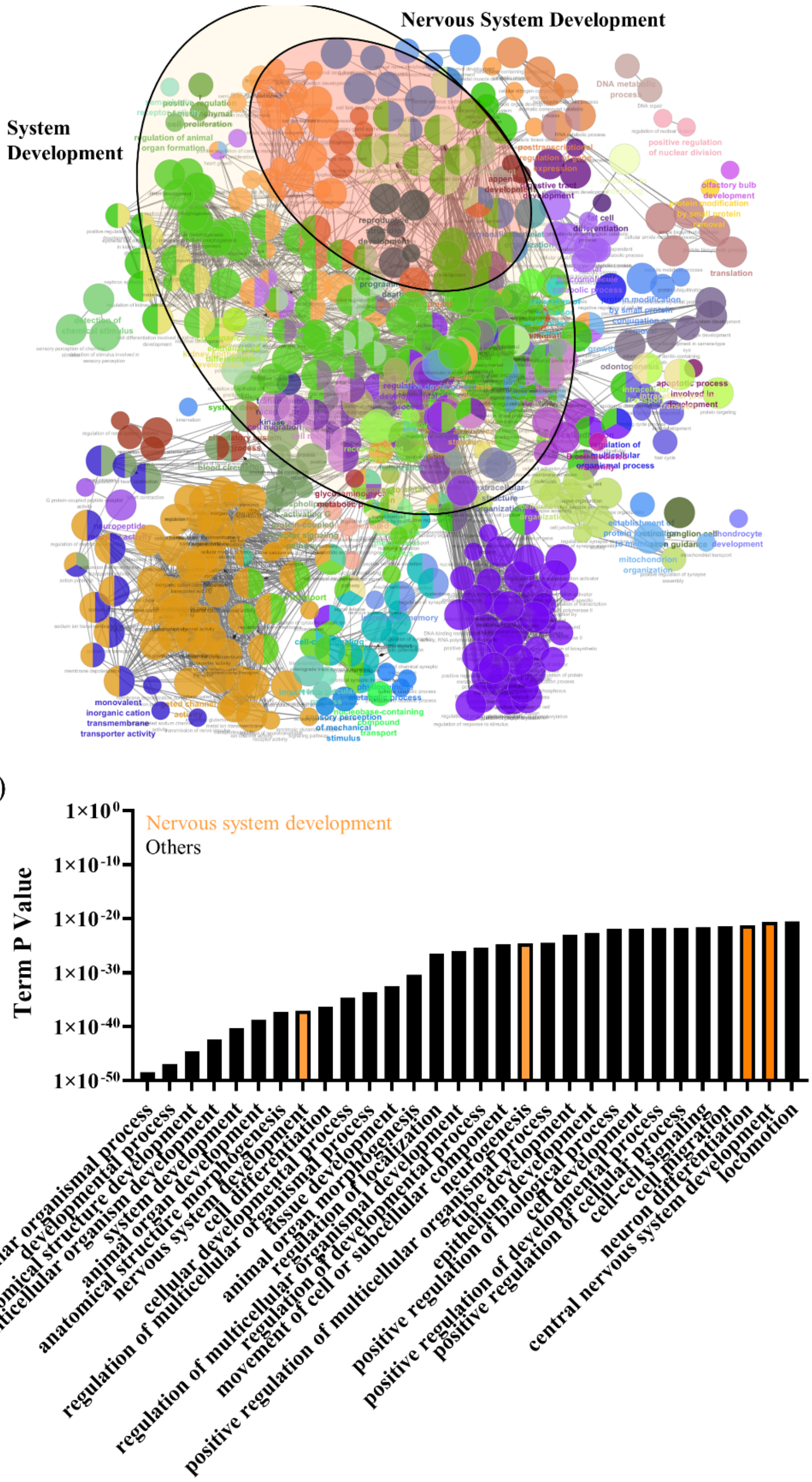

Supplementary Figure S6: (**a**) Gene ontology network map for differentially expressed genes in CHLA266 human ATRT cells treated with 10nM panobinostat for 21 days (FDR< 0.05; LogFc<-2 or >2; pV<0.05). (**b**) Most significant functional groups associated with differentially expressed genes.

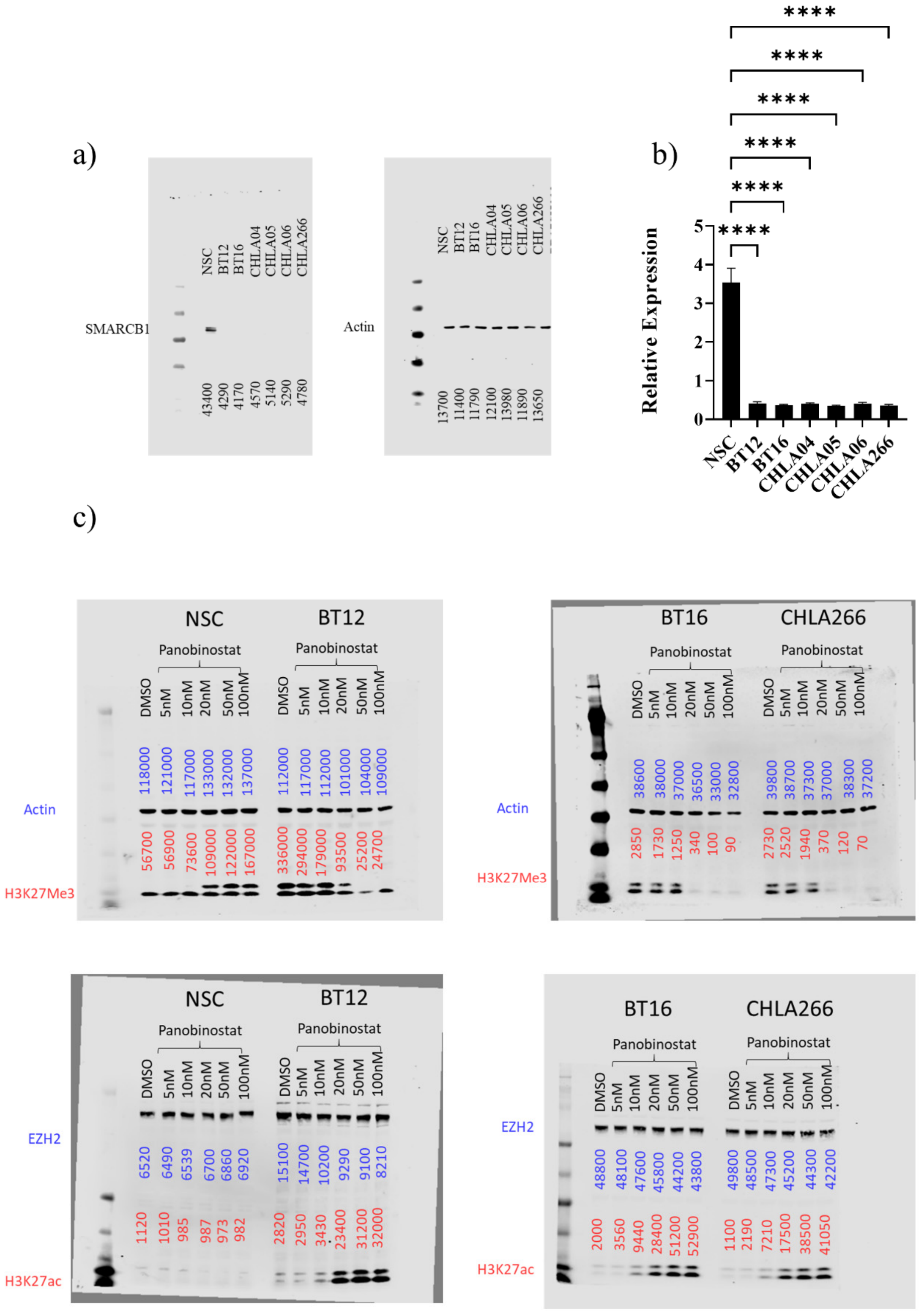

Figure S7: Full western blot images including densitometry values. (**a**) Figure 1a. (**b**) Densitometry analysis of SMARCB1 expression in ATRT (*n*=3; mean SEM; ****, p <0.0001). (**c**) Figure 1c
